# *Shank3*-mutant mice lacking exon 9 show altered excitation/inhibition balance, enhanced rearing, and spatial memory deficit

**DOI:** 10.3389/fncel.2015.00094

**Published:** 2015-03-19

**Authors:** Jiseok Lee, Changuk Chung, Seungmin Ha, Dongmin Lee, Do-Young Kim, Hyun Kim, Eunjoon Kim

**Affiliations:** ^1^Department of Biological Sciences, Korea Advanced Institute of Science and TechnologyDaejeon, South Korea; ^2^Department of Anatomy and Division of Brain Korea 21, Biomedical Science, College of Medicine, Korea UniversitySeoul, South Korea; ^3^Center for Synaptic Brain Dysfunctions, Institute for Basic ScienceDaejeon, South Korea

**Keywords:** autism, Shank3, E/I ratio, hyperactivity, memory, synaptic transmission

## Abstract

Shank3 is a postsynaptic scaffolding protein implicated in synapse development and autism spectrum disorders. The *Shank3* gene is known to produce diverse splice variants whose functions have not been fully explored. In the present study, we generated mice lacking *Shank3* exon 9 (*Shank3*^Δ*9*^ mice), and thus missing five out of 10 known Shank3 splice variants containing the N-terminal ankyrin repeat region, including the longest splice variant, Shank3a. Our X-gal staining results revealed that Shank3 proteins encoded by exon 9-containing splice variants are abundant in upper cortical layers, striatum, hippocampus, and thalamus, but not in the olfactory bulb or cerebellum, despite the significant Shank3 mRNA levels in these regions. The hippocampal CA1 region of *Shank3*^Δ*9*^ mice exhibited reduced excitatory transmission at Schaffer collateral synapses and increased frequency of spontaneous inhibitory synaptic events in pyramidal neurons. In contrast, prelimbic layer 2/3 pyramidal neurons in the medial prefrontal cortex displayed decreased frequency of spontaneous inhibitory synaptic events, indicating alterations in the ratio of excitation/inhibition (E/I ratio) in the *Shank3*^Δ*9*^ brain. These mice displayed a mild increase in rearing in a novel environment and mildly impaired spatial memory, but showed normal social interaction and repetitive behavior. These results suggest that ankyrin repeat-containing Shank3 splice variants are important for E/I balance, rearing behavior, and spatial memory.

## Introduction

Shank represents a family of synaptic scaffolding proteins with three known members: Shank1/ProSAP3, Shank2/ProSAP1, and Shank3/ProSAP2 (Sheng and Kim, 2000, 2011; Sheng and Sala, 2001; Ehlers, 2002; Sheng and Hoogenraad, 2007; Verpelli et al., 2012). Shank proteins are thought to form postsynaptic multi-protein complexes together with other scaffolds, receptors, and signaling molecules, serving to couple receptor activation with postsynaptic signaling (Boeckers et al., 1999; Naisbitt et al., 1999; Tu et al., 1999; Hayashi et al., 2009).

Genetic variations of *SHANK3*, together with those for *SHANK2*, are strongly associated with diverse brain dysfunctions, including autism spectrum disorders (ASDs) Phelan-McDermid syndrome, schizophrenia, and intellectual disability (Bonaglia et al., 2001, 2011; Durand et al., 2007; Moessner et al., 2007; Berkel et al., 2010; Gauthier et al., 2010; Hamdan et al., 2011; Leblond et al., 2012, 2014; Boccuto et al., 2013; Guilmatre et al., 2014). Mouse genetic studies also support a role for Shank3 in diverse brain functions, including social interaction, social communication, repetitive behavior, cognitive functions, and anxiety (Bozdagi et al., 2010; Peca et al., 2011; Wang et al., 2011; Schmeisser et al., 2012; Yang et al., 2012; Han et al., 2013; Jiang and Ehlers, 2013; Kouser et al., 2013; Wohr, 2014), which, together with molecular and cell biological studies of Shank3, have suggested candidate mechanisms underlying Shank3-related brain disorders and potential strategies for treating them (Boeckers et al., 2002; Grabrucker et al., 2011a,b, 2014; Arons et al., 2012; Verpelli et al., 2012; Betancur and Buxbaum, 2013; Bozdagi et al., 2013; Jiang and Ehlers, 2013; Mameza et al., 2013; Carbonetto, 2014; Epstein et al., 2014; Guilmatre et al., 2014; Wang et al., 2014a,b; Zhu et al., 2014).

Shank3, like other Shank proteins, contain diverse domains, including (from the N-terminus) ankyrin repeats, an SH3 domain, a PDZ domain, a proline-rich region and a SAM domain, which mediate the interactions with other synaptic proteins. Diverse ASD-associated *Shank3* mutations are likely to disrupt the structure and function of these domains (Arons et al., 2012; Durand et al., 2012; Mameza et al., 2013), although little is known about how these mutations induce specific defects in protein and synapse structure/function, or deficits in neural circuits and brain functions.

Importantly, alternative splicing in the *Shank3* gene has been suggested to produce a large number of splice variants (Lim et al., 1999; Maunakea et al., 2010; Waga et al., 2014; Wang et al., 2014b). Specifically, the mouse *Shank3* gene contains a total of 22 exons, that together encode a full-length protein of 1730 amino acids (aa). Alternative translational start/stop and splicing insertion/deletion sites are predicted to produce a total of 10 splice variants of the Shank3 protein (Wang et al., 2014b). Five out of the 10 Shank3 splice variants, including the longest one (Shank3a), share the ankyrin repeats, suggesting that this region is important for the function of Shank3 proteins. Ankyrin repeats are thought to function as a protein-recognition domain that interacts with proteins including α-fodrin and Sharpin (in the case of Shank3) (Bockers et al., 2001; Lim et al., 2001). By forming a superspiral structure, this domain is also thought to act as a “molecular spring” (Lee et al., 2006).

Previous studies have reported transgenic mice carrying various deletions of *Shank3* exons encoding the ankyrin repeats (exons 4–9), demonstrating that these mice display a range of synaptic and ASD-related impairments (Bozdagi et al., 2010; Peca et al., 2011; Wang et al., 2011; Yang et al., 2012). Given that *Shank3* mutations are located on different parts of the N-terminal region, including the ankyrin repeats (Leblond et al., 2014), and each variation is likely to contribute differentially to the structure and function of the protein and, by extension, to the types and severity of *SHANK3*-related ASD symptoms, a comparison of multiple lines of mice lacking different parts of the exon 4–9 region might prove informative.

In the present study, we generated a line of transgenic mice lacking exon 9 of the *Shank3* gene encoding the last ankyrin repeat. X-gal staining showed that ankyrin repeat containing splice variants are widely expressed in various forebrain regions but not in the olfactory bulb or cerebellum, despite the fact that Shank3 mRNAs are abundant in these regions. The *Shank3*^Δ*9*^ hippocampus showed reduced excitatory synaptic transmission at Schaffer collateral-CA1 synapses but increased frequency of spontaneous inhibitory synaptic events. This contrasted with the decreased frequency of spontaneous inhibitory synaptic events in layer 2/3 pyramidal neurons in the prelimbic region of the medial prefrontal cortex (mPFC), suggesting alterations in the excitation/inhibition (E/I) ratio in different brain regions. Behaviorally, *Shank3*^Δ*9*^ mice showed normal social interaction and repetitive behavior, but exhibited a mild increase in rearing in a novel environment and mildly impaired spatial memory, suggesting that exon 9-containing Shank3 splice variants may be important for rearing behavior and spatial memory.

## Materials and methods

### Generation of *Shank3*^Δ*9*^ mice

Mouse ES cell line with *Shank3* exon 9 floxed was purchased from the Knockout Mouse Project (KOMP) repository (Project name: CSD48829). ES cells were injected into C57BL/6N blastocysts to produce chimeric mice. Chimeric mice were crossed with wild-type C57BL/6N to produce F1 mice with the floxed allele. F1 mice were crossed with Protamine-Flp mice to remove the β-gal-Neo cassette (F2). F2 mice were crossed with Protamine-Cre mice, and the progeny F3 mice were crossed with wild-type to obtain the *Shank3*^Δ*9*^ allele (F4). All mice used in experiments were obtained by heterozygous mating (+/Δ9 × +/Δ9). Mice were bred and maintained according to the Requirements of Animal Research at KAIST, and all procedures were approved by the Committee of Animal Research at KAIST (KA2012-19).

### Genotyping PCR

The following primers were used to detect wild-type, floxed, and Δ9 alleles. Forward 1: GAGTTTTGCAGGAGTTGAAGGT, Reverse 1: CTCATTTCCTGTGTCAGCAGAG, Reverse 2: CAAGGAGCTCGTAACCAAGC. Forward 1 + Reverse 1 primers were used to detect the wild-type (756 bp) or floxed (855 bp) allele. Forward 1 + Reverse 2 primers were used for the Δ9 allele (995 bp).

### RT-PCR

Mouse brains (P12) were sectioned using Brain Matrix, and random punches from the slices were processed with Qiagen RNeasy Plus Mini Kit to obtain RNA. cDNAs were synthesized from the obtained RNAs using Enzynomics M-MLV cDNA synthesis kit. Following primers were used to detected wild-type (979 bp) and Δ9 (912 bp) alleles. Forward: CTACGGGCTA TTCCAGCCTC CCTC, Reverse: GTTGATATCA CTGGCTGAGCGCTG.

### Antibodies

Shank3 guinea pig polyclonal antibodies were raised using keyhole limpet hemocyanin coupled with peptides mimicking amino acids 192–221, 529–558, 1289–1318 of the mouse Shank3 protein (NCBI NP_067398.2).

### Western blot

Specific mouse brain regions (3–6 months) were placed in ice-cold section buffer (212 mM sucrose, 25 mM NaHCO_3_, 5 mM KCl, 1.25 mM NaH_2_PO_4_, 10 mM glucose, 1.2 mM ascorbic acid, 2 mM pyruvic acid, 3.5 mM MgSO_4_, 0.5 mM CaCl_2_) and homogenized by motorized tissue grinder in ice-cold homogenization buffer (0.32 M sucrose, 10 mM HEPES, 2 mM EDTA, 2mM EGTA, protease inhibitors and phosphatase inhibitors). After immunoblotting, fluorescent secondary antibody signals were detected using *Odyssey*^®^ *Fc Dual Mode Imaging System.*

### *In situ* hybridization

*In situ* hybridization was performed essentially as previously described (Kim et al., 2004). The whole bodies (embryonic days 16 and 18) and brains (postnatal days 7, 14, and 21, and week 6) of mice were extracted and rapidly frozen in isopentane prechilled with dry ice, and the frozen sections were cut (12 μm thick) and thaw-mounted onto gelatin-coated slides and fixed in 4% paraformaldehyde. Hybridization probe specific for mouse Shank3 mRNA was prepared using the following region: nt 118–869 of *Shank3* (NM_021423.3). Antisense riboprobes were generated using ^35^S-UTP and the Riboprobe system (Promega).

### X-gal staining

Mice (6–7 weeks) were perfused transcardially with 4% paraformaldehyde. Brains were removed and sectioned into 250 μm slices. Slices were incubated in staining solution (5 mM K_3_Fe(CN)_6_, 5 mM K_4_Fe(CN)_6_•3H_2_O, 2 mM MgCl_2_, 0.01% deoxycholate, 1 mg/mL X-gal, 0.02% NP-40 in PBS) for 1 h 30 min at room temperature. Stained slices were washed four times with PBS and mounted for light microscopy.

### Field recording

Mice (P19–25) were anesthetized with diethyl ether, brains were removed and sagittal sections (400 μm) including hippocampus were prepared in ice-cold section buffer (in mM: 212 sucrose, 25 NaHCO_3_, 5 KCl, 1.25 NaH_2_PO_4_, 10 glucose, 1.2 ascorbic acid, 2 pyruvic acid, 3.5 MgSO_4_, 0.5 CaCl_2_). Slices were maintained in artificial cerebrospinal fluid (in mM: 124 NaCl, 25 NaHCO_3_, 10 glucose, 2.5 KCl, 1 NaH_2_PO_4_, 2.5 CaCl_2_, 1.25 MgSO_4_) bubbled with 95% O_2_ and 5% CO_2_ at room temperature. The stratum radiatum of hippocampal CA1 field was stimulated and recorded with glass pipettes filled with ACSF. Stimulus was given every 20 s to monitor the baseline responses. Stimulator: A-M Systems Model 2100, amplifier: Axon CNS MultiClamp 700B, digitizer: Axon CNS Digidata 1440A, data monitoring and recording: Clampex 10.3.1.5. After baselines were stabilized, a single 100 HZ stimulation for 1 s was given for LTP induction.

### Whole cell recording

Mice (P19–22 for CA1 mEPSC, P23–27 for CA1 mIPSC, and P39–54 for mPFC mEPSC and mIPSC) were anesthetized with diethyl ether, brains were removed and sagittal sections (300 μm) including hippocampus or coronal sections (300 μm) including mPFC were prepared in ice-cold section buffer (in mM: 212 sucrose, 25 NaHCO_3_, 5 KCl, 1.25 NaH_2_PO_4_, 10 D-glucose, 1.2 L-ascorbic acid, 2 Na-pyruvate, 3.5 MgSO_4_, 0.5 CaCl_2_). Slices were maintained in artificial cerebrospinal fluid (in mM: 124 NaCl, 25 NaHCO_3_, 10 glucose, 2.5 KCl, 1 NaH_2_PO_4_, 2.5 CaCl_2_, 1.25 MgSO_4_) bubbled with 95% O_2_ and 5% CO_2_ at room temperature. For mEPSC experiment, ACSF contained tetrodotoxin (0.5 μM) and picrotoxin (60 μM). CA1 or mPFC pyramidal cells were voltage-clamped and recorded with glass pipettes filled with internal solution containing (in mM): 117 CsMeSO_4_, 10 TEA-Cl, 8 NaCl, 10 HEPES, 5 QX-314-Cl, 4 Mg-ATP, 0.3 Na-GTP, 10 EGTA, with pH 7.25, 295 mOsm. For mIPSC experiment, ACSF contained tetrodotoxin (0.5 μM), NBQX (10 μM), and AP∨ (50 μM). CA1 or mPFC pyramidal cells were voltage-clamped and recorded with glass pipettes filled with internal solution containing (in mM): 115 CsCl, 10 TEA-Cl, 8 NaCl, 10 HEPES, 5 Qx-314-Cl, 4 Mg-ATP, 0.3 Na-GTP, 10 EGTA with pH 7.35, 295 mOsm. Amplifier: Axon CNS MultiClamp 700B, digitizer: Axon CNS Digidata 1440A, data monitoring and recording: Clampex 10.4.

### Three-chamber social interaction test

A white acrylic box (60 cm W × 40 cm D × 20 cm H) partitioned into three chambers was used. First, mice (2–4 months) were allowed to freely explore the chambers for 10 min. Next, a stranger mouse (S1) was put in a small cage in one side chamber, and an object (O) was put in a cage in another side chamber. Mice were then allowed to explore freely for 10 min. Next, the object (O) was replaced with another novel stranger mouse (S2), and mice were allowed to freely explore either the familiar mouse (S1) or the novel mouse (S2) for 10 min. Exploration time was defined as time spent in sniffing the cage containing either O, S1, or S2. Preference index was calculated with exploration time. O vs. S1 preference index = (S1 − O)/(S1 + O) × 100. S1 vs. S2 preference index = (S2 − S1)/(S2 + S1) × 100.

### Separation-induced pup ultrasonic vocalization

Pups (P4–10) were placed in a glass bowl inside a Styrofoam box in a sound-proof booth. A recording microphone was placed 20 cm above the pup. Separation-induced ultrasonic vocalization was recorded for 3 min using Avisoft Ultrasoundgate (Model 116Hb) system. Recorded sound files were analyzed using Avisoft SASLab Pro software. Sound files were transformed into spectrograms, and the numbers of USV calls were counted automatically by the software.

### Laboras™ monitoring of 72-h movements

Locomotion and various behaviors of mice were recorded and analyzed using Laboratory Animal Behavior Observation Registration and Analysis System (LABORAS™) by *Metris.* Mice (2–4 months) were put into LABORAS recording cages where recordings were conducted for 72 consecutive hours.

### Open field test

Mice (2–4 months) were put in a white acrylic box (40 cm W × 40 cm D × 40 cm H), and their horizontal locomotion was recorded by a video camera from above for 60 min. The brightness of the open field was 120–130 lux. Recorded video was automatically analyzed using Noldus EthoVision XT 10 software. Center zone was defined as the center 4 × 4 squares when the field was subdivided into 6 × 6 squares.

### Morris water maze

A circular tank with 120 cm diameter was used. A platform with 10 cm diameter was placed in one of the quadrants. Water was filled so that the platform was 1 cm beneath the water surface. White paint was used to make the water opaque. Water temperature was kept at 22–24°C. Visual cues of various shapes were hung up around the tank. For each trial, mice (2–4 months) were allowed to search for the hidden platform for 1 min. If they found the platform before 1 min, they were allowed to stay on it for 15 s. If they did not find the platform before 1 min, they were guided by hand to the platform, and allowed to stay on it for 15 s. Each mouse did three trials per day. Three trials were averaged for calculating the escape latency of each mouse each day. In the probe test, the platform was removed, and mice were allowed to search the platform for 1 min. Twenty four hours after the probe test, the platform was re-located to the opposite position, and mice went through learning trials and the probe test for reversal learning test. Time spent in each quadrant, swim speed, and number of platform area crossings were analyzed automatically using Noldus EthoVision XT 10 software.

### Novel object recognition test

Two identical cylinder-shaped plastic objects were placed apart from each other at the midline of a white acrylic box (40 cm W × 40 cm D × 40 cm H). Mice (2–4 months) were allowed to freely explore the objects for 10 min. After 24 h, one of the objects was replaced by a novel, rectangular prism-shaped metallic object. Mice were again allowed to freely explore the objects for 10 min. The illumination was 120–130 lux. Exploration time was defined as the time mice spent contacting and sniffing the familiar (*F*) or novel (*N*) object. Preference index was calculated using exploration time. *N* vs. F preference index = *N*/(*N* + *F*) × 100. Locomotion was analyzed automatically using Noldus EthoVision XT 10 software.

### Statistics

Details on the statistical results are described in Supplementary Table 1.

## Results

### Generation and characterization of *Shank3*^Δ*9*^ mice

**Figure 1:**
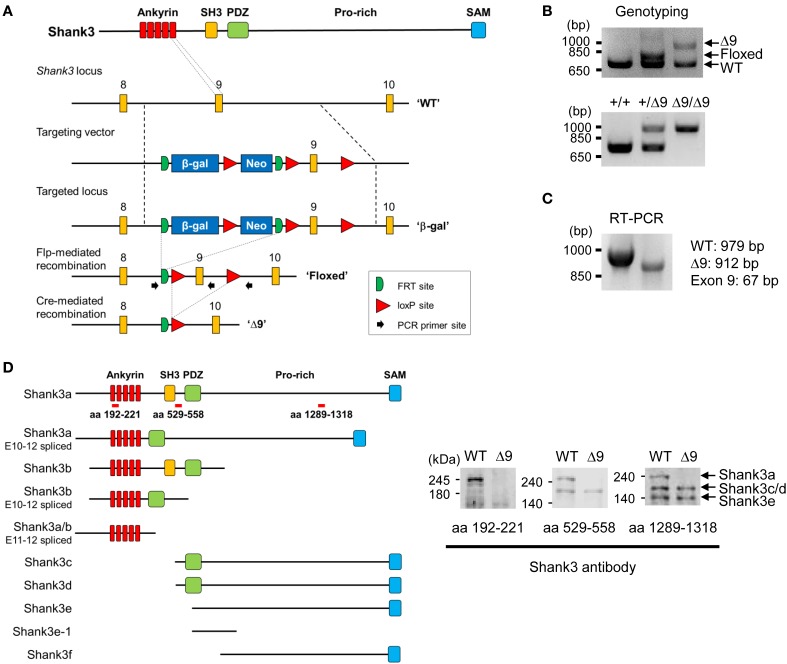
**Generation and characterization of *Shank3*^Δ*9*^ mice. (A)** Targeting the *Shank3* locus in mice and removal of exon 9 by Cre-mediated recombination. Ankyrin, ankyrin repeats; SH3, src homology 3 domain; PDZ, PSD-95, Dlg, ZO-1 domain; Pro-rich, proline-rich region; SAM, sterile alpha motif; β-gal, β-galactosidase; WT, wild-type. **(B)** Genotyping PCR of the targeted locus. Δ9, *Shank3*^Δ*9*^. **(C)** RT-PCR of WT and *Shank3*^Δ*9*^ mouse brains showing the removal of exon 9 at the mRNA level. **(D)** Immunoblotting of Shank3 splice variants in WT and *Shank3*^Δ*9*^ brains by three different antibodies. Targeted regions of the antibodies are indicated by red bars on the recently reported 10 Shank3 splice variants (Wang et al., 2014b). Note that the longest isoform (Shank3a band) is undetectable in the *Shank3*^Δ*9*^ brain.

*Shank3*^Δ*9*^ mice were generated by introducing a construct containing a floxed exon 9 encoding the last ankyrin repeat of the protein (Figure 1A). Removal of exon 9 by Cre-mediated recombination led to a frameshift and premature stop in the coding region. The Δ9 allele was confirmed by genomic PCR (Figure 1B) and RT-PCR (Figure 1C).

In order to determine which splice variants of Shank3 were eliminated in this mouse line, we used three antibodies that specifically target different regions of Shank3: the N-terminus, the middle region (SH3-PDZ), and the C-terminal proline-rich region (Figure 1D). Western blot analyses of brain lysates using these antibodies revealed three major proteins bands of ~240, ~190, and ~140 kDa in wild-type (WT) mice, which we refer to hereafter as Shank3a, Shank3c/d, and Shank3e, respectively, according to the reported nomenclature of Shank3 (Wang et al., 2014b) (Figure 1D).

Notably, in the *Shank3*^Δ*9*^ brain, only Shank3a (longest variant), was clearly undetectable by all three antibodies, whereas Shank3c/d and Shank3e remained intact (Figure 1D). These results suggest that *Shank3*^Δ*9*^ mice lack at least the longest Shank3 splice variant, and likely other smaller splice variants containing ankyrin repeats.

### Expression patterns of ankyrin repeat-containing variants of Shank3 mRNAs and proteins

**Figure 2:**
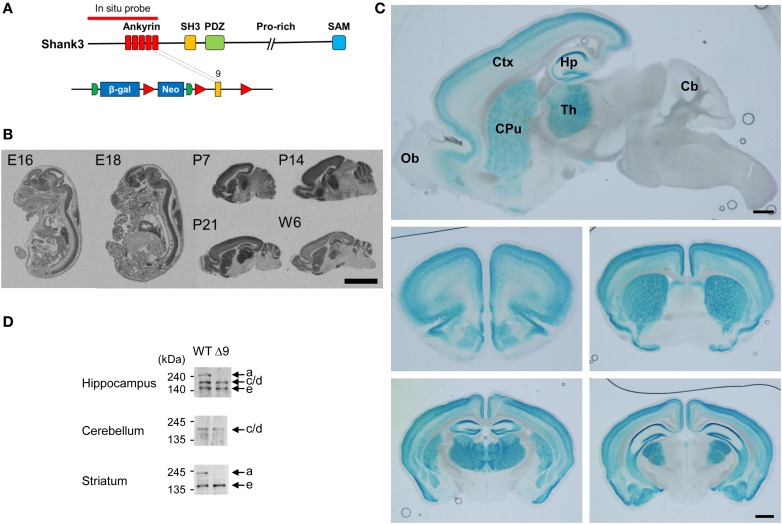
**Expression patterns of ankyrin repeat-containing variants of Shank3 mRNAs and proteins. (A)** Locations of the *in situ* hybridization probe and β-galactosidase insertion. **(B)** Distribution patterns of ankyrin repeat-containing *Shank3* mRNA variants in mouse embryonic and postnatal brain (sagittal) sections, as revealed by *in situ* hybridization. Scale bar, 5 mm. E, embryonic; P, postnatal day; W6, postnatal week 6. **(C)** Ankyrin repeat-containing Shank3 protein variants, as revealed by X-gal staining of sagittal (top) and coronal (middle and bottom) *Shank3*^+/β-*gal*^ brain sections (6–7 weeks). Ob, olfactory bulb; Ctx, cortex; Hp, hippocampus; Th, thalamus; CPu, striatum; Cb, cerebellum. Scale bar, 1 mm. **(D)** Differential expression of Shank3 protein variants in different brain regions, as revealed by immunoblot analysis of WT and *Shank3*^Δ*9*^ brain lysates (3–6 months) with the Shank3 antibody (aa 1289–1318).

We first determined the brain regions in which exon 9-containing *Shank3* transcripts are expressed by *in situ* hybridization using a probe encompassing the exon 1–9 region (Figure 2A). We found strong signals in brain and spinal cord regions at embryonic days 16 and 18 (Figure 2B). At postnatal days (P) 7, 14, and 21 and week 6, signals were detected in the olfactory bulb, cortex, striatum, hippocampus, thalamus, and cerebellum (Figure 2B).

We next examined the distribution patterns of Shank3 protein variants encoded by exon 9-containing splice variants by X-gal staining (Figure 2A), which would detect all Shank3 N-terminal fragments fused to β-galactosidase. These signals were strongly detected in the cortex, striatum, hippocampus, and thalamus of 6–7 week-old mice, a pattern similar to that observed by *in situ* hybridization (Figure 2C; Supplementary Figure 1; Table 1). In sharp contrast to the *in situ* hybridization results, however, these signals were essentially undetectable in the cerebellum and olfactory bulb (Figure 2C).

**Table 1:** **Summary of the X-gal staining results in *Shank3*^+/β-*gal*^ brain slices**.

**Brain regions**	**Expression**
Main olfactory bulb	−
Cerebral cortex	
Layer 1	−
Layer 2/3	+++
Layer 4	+++
Layer 5	+
Layer 6	++
Hippocampus	
Dentate gyrus	+++
CA3	++
CA1	+++
Habenula	−
Thalamus	+++
Reticular thalamic nucleus	−
Basal ganglia	
Caudate putamen	+++
Globus pallidus	−
Substantia nigra	−
Amygdala	++
Hypothalamus	−
Ventral tegmental area	−
Cerebellum	−

Consistent with this difference, immunoblot analyses of brains at 3–6 months revealed that the longest Shank3 protein variant (Shank3a), which contains the ankyrin repeat region, was undetectable in the cerebellum, but was clearly visible in the hippocampus and striatum (Figure 2D), similar to recently reported results (Wang et al., 2014b).

Notably, X-gal staining revealed differential expression patterns of ankyrin repeat-containing Shank3 protein variants within a specific brain region. For instance, in the hippocampus, signals were stronger in the CA1 and dentate gyrus (DG) than in CA3 (Figure 2C; Supplementary Figure 1, pp. 1–3), and signals in cortical layers 2–4 were stronger than those in layers 5 and 6 (Supplementary Figure 2).

### Reduced excitatory transmission and increased mIPSC frequency in the *Shank3*^Δ*9*^ hippocampus

**Figure 3:**
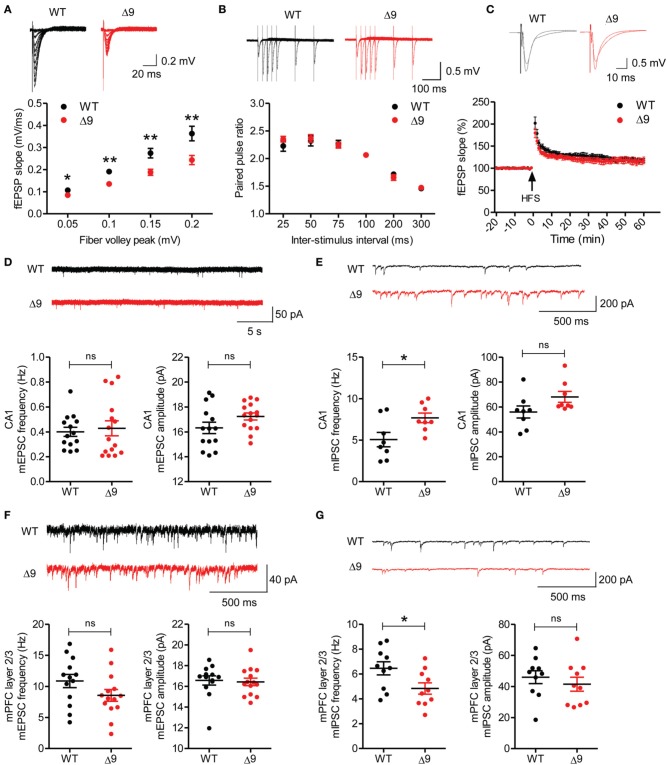
**Reduced excitatory transmission and increased mIPSC frequency in the *Shank3*^Δ*9*^ hippocampus, and decreased mIPSC frequency in the *Shank3*^Δ*9*^ mPFC. (A)** Reduced excitatory synaptic transmission at *Shank3*^Δ*9*^ hippocampal SC-CA1 synapses (P19–25), as revealed by plots of fEPSP slopes against fiber volley amplitudes (input–output). *Inset*, representative traces. *N* = 9 cells from three mice for WT and Δ9. **(B)** Normal paired-pulse facilitation at *Shank3*^Δ*9*^ SC-CA1 synapses (P19–25). *N* = 9 cells from three mice for WT and Δ9. *Inset*, representative traces. **(C)** Normal LTP induced by high-frequency stimulation (HFS) at *Shank3*^Δ*9*^ SC-CA1 synapses (P21–24). *Inset*, representative trace before and after stimulus. *N* = 8 slices from four mice (WT), seven slices from three mice (*Shank3*^Δ*9*^). **(D)** Normal frequency and amplitude of mEPSCs in *Shank3*^Δ*9*^ CA1 pyramidal cells (P19–22). *N* = 14 cells from three mice (WT), 15 cells from three mice (*Shank3*^Δ*9*^). **(E)** Increased frequency and normal amplitude of mIPSCs in *Shank3*^Δ*9*^ CA1 pyramidal cells (P23–27). *N* = 8 cells from four mice (WT), eight from three mice (*Shank3*^Δ*9*^). **(F)** Normal frequency and amplitude of mEPSCs in layer 2/3 pyramidal neurons in the prelimbic region of the mPFC in *Shank3*^Δ*9*^ mice (P39–45). *N* = 13 cells from three mice (WT), 14 cells from three mice (*Shank3*^Δ*9*^). **(G)** Decreased frequency and normal amplitude of mIPSCs in *Shank3*^Δ*9*^ mPFC prelimbic layer 2/3 pyramidal cells (P39–54). *N* = 10 cells from four mice (WT), 10 from three mice (*Shank3*^Δ*9*^). ^*^P < 0.05, ^**^P < 0.01, ns, not significant, Student's *t*-test. Data represent mean ± standard error.

We next assessed whether *Shank3* exon-9 deficiency leads to any changes in synaptic function. Excitatory transmission in *Shank3*^Δ*9*^ Schaffer collateral-CA1 pyramidal (SC-CA1) synapses (P19–25) were significantly decreased relative to those at WT synapses, as measured by plots of field excitatory postsynaptic potential (fEPSP) slopes against fiber volley amplitudes (input-output) (Figure 3A). Paired pulse ratios at SC-CA1 synapses (P19–25) were not different between genotypes (Figure 3B), suggesting that presynaptic release probability was not changed. In addition, long-term potentiation (LTP) at SC-CA1 synapses (P21–24) induced by high-frequency stimulation (100 Hz, 1 s) was comparable between *Shank3*^Δ*9*^ and WT synapses (Figure 3C).

*Shank3*^Δ*9*^ CA1 pyramidal cells (P19–22) showed normal amplitude and frequency of miniature excitatory postsynaptic currents (mEPSCs) compared with those of WT mice (Figure 3D). Notably, the frequency, but not the amplitude, of miniature inhibitory postsynaptic currents (mIPSCs) was significantly increased in *Shank3*^Δ*9*^ CA1 pyramidal cells (P23–27) relative to WT neurons (Figure 3E). These results suggest that Shank3 exon-9 deletion leads to an increase in inhibitory synaptic input to CA1 neurons, and, together with the decreased excitatory synaptic transmission, suggests a reduction of the E/I ratio in the CA1 region.

### Decreased mIPSC frequency but normal mEPSCs in the *Shank3*^Δ*9*^ mPFC

We additionally measured synaptic transmission in the medial prefrontal cortex (mPFC), a brain region implicated in ASDs. mEPSCs measured in layer 2/3 pyramidal neurons in the prelimbic area of the mPFC in *Shank3*^Δ*9*^ mice (P39–54) were normal in both frequency and amplitude, when compared with WT neurons (Figure 3F). In contrast, these cells displayed decreased mIPSC frequency, although the mIPSC amplitude was normal (Figure 3G). These results indicate that *Shank3*^Δ*9*^ mPFC pyramidal neurons display increased E/I ratio, and, together with the results from the hippocampus, suggest that *Shank3* exon-9 deletion leads to distinct alterations of the E/I ratio in different brain regions.

### *Shank3*^Δ*9*^ mice do not show autistic-like behavior

**Figure 4:**
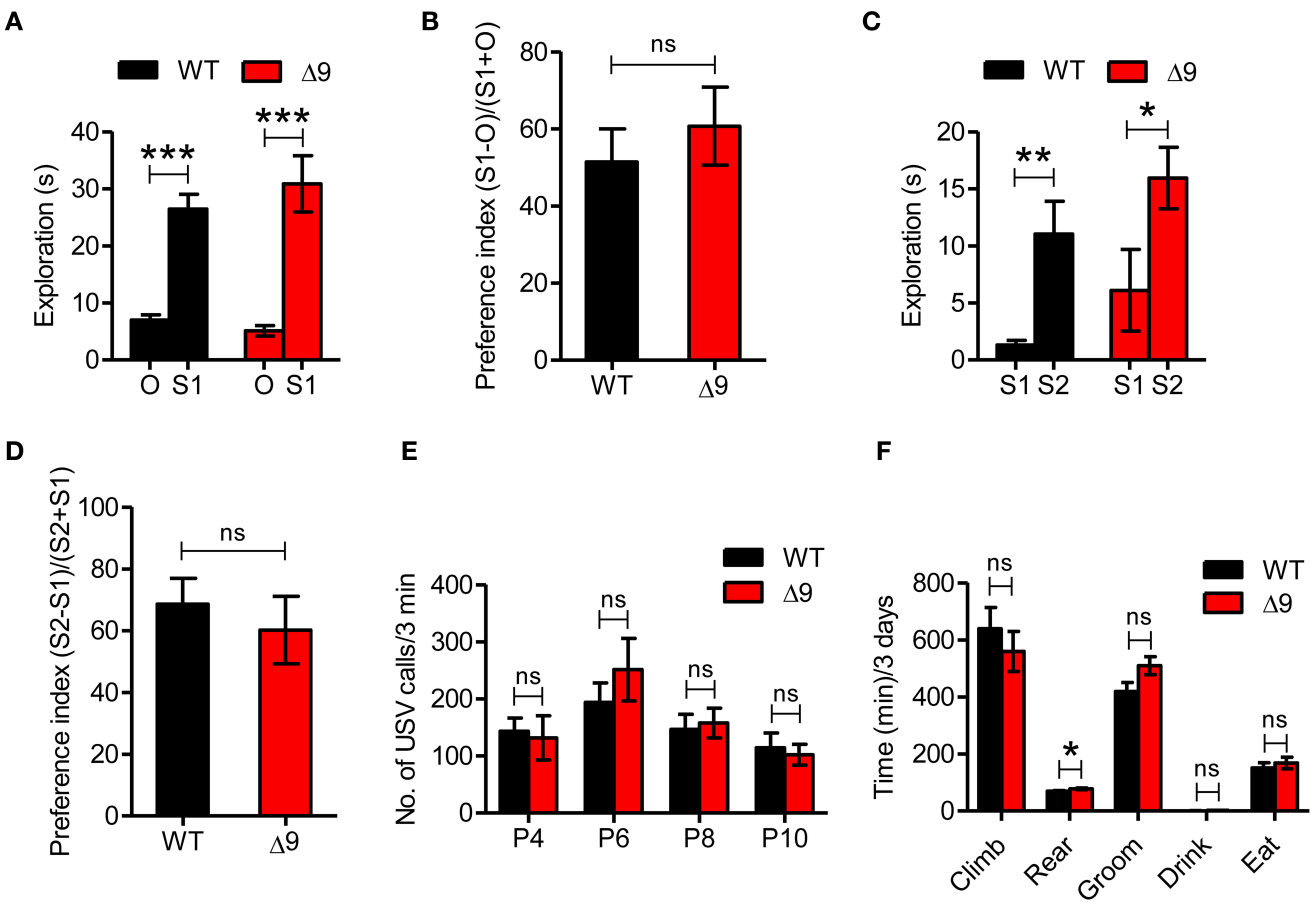
***Shank3*^Δ*9*^mice do not show autistic-like behavior. (A, B)** *Shank3*^Δ*9*^ mice (2–4 months) show normal exploration of a stranger mouse (S1) vs. an inanimate object (O) in the three-chamber social interaction test, as shown by time spent in exploration **(A)** and social preference index **(B)**. *N* = 23 (WT), 19 (*Shank3*^Δ*9*^). **(C, D)** WT and *Shank3*^Δ*9*^ mice show similar preference toward a novel mouse (S2) over a familiar mouse (S1) in the three-chamber social interaction test. *N* = 8 (WT), 10 (*Shank3*^Δ*9*^), as shown by time spent in exploration **(C)** and social preference index **(D)**. **(E)** *Shank3*^Δ*9*^ pups (P4–10) emit normal numbers of USVs when separated from their mother, compared with WT mice. *N* = 11 (WT), 9 (*Shank3*^Δ*9*^). **(F)** *Shank3*^Δ*9*^ mice do not show repetitive behaviors except for a small increase in rearing, as determined by behavioral monitoring for three consecutive days with normal light-dark cycles. *N* = 9 (WT), 11 (*Shank3*^Δ*9*^). ^*^P < 0.05, ^**^P < 0.01, ^***^P < 0.001, ns, not significant, Student's *t*-test. Data represent mean ± standard error.

Given the well-known association of Shank3 with ASDs, we first tested autistic-like behaviors in *Shank3*^Δ*9*^ mice. In the three-chamber social interaction test, both WT and *Shank3*^Δ*9*^ mice (2–4 months) showed a preference for exploring the stranger mouse compared with an inanimate object (Figures 4A,B; Table 2; Supplementary Table 1). When the object was replaced with another novel mouse, both WT and *Shank3*^Δ*9*^ mice preferred the novel mouse over the familiar mouse (Figures 4C,D). These results suggest that *Shank3*^Δ*9*^ mice display normal social interaction and social novelty recognition.

**Table 2:** **Summary of the behavioral results in *Shank3*^Δ*9*^ mice**.

**Behavior category**	**Assay**	**Result**
Social behavior	Three-chamber social interaction (object vs. stranger 1)	Normal
Social novelty recognition	Three-chamber social interaction (stranger 1 vs. stranger 2)	Normal
Social communication	Separation-induced pup ultrasonic vocalization	Normal
Repetitive behavior	Seventy two-hour behavior monitoring	Normal except a small increase in rearing at the beginning of cage habituation
Locomotor activity	Seventy two-hour behavior monitoring	Normal
	Open field	Normal
Anxiety-like behavior	Open field	Normal
Spatial learning and memory	Morris water maze	Largely normal, except for a decrease in the number of platform crossing
Object recognition memory	Novel object recognition	Normal

We next measured ultrasonic vocalization (USV) in *Shank3*^Δ*9*^ mice (P4–10), using separation of pups from their mother to induce USVs. These tests showed that *Shank3*^Δ*9*^ mice emitted normal levels of separation-induced USVs at postnatal days 4, 6, 8, and 10 (Figure 4E), suggesting the absence of USV defects.

Repetitive behavior is another key autistic-like phenotype. When *Shank3*^Δ*9*^ mice (2–4 months) were placed in a cage environment distinct from their home cages (Laboras cage™) and their various behaviors were monitored for 72 consecutive hours with normal light-dark cycles, they showed no repetitive behaviors such as grooming, but did display a small increase in rearing behavior (Figure 4F).

### *Shank3*^Δ*9*^ mice show increased rearing in a novel environment

**Figure 5:**
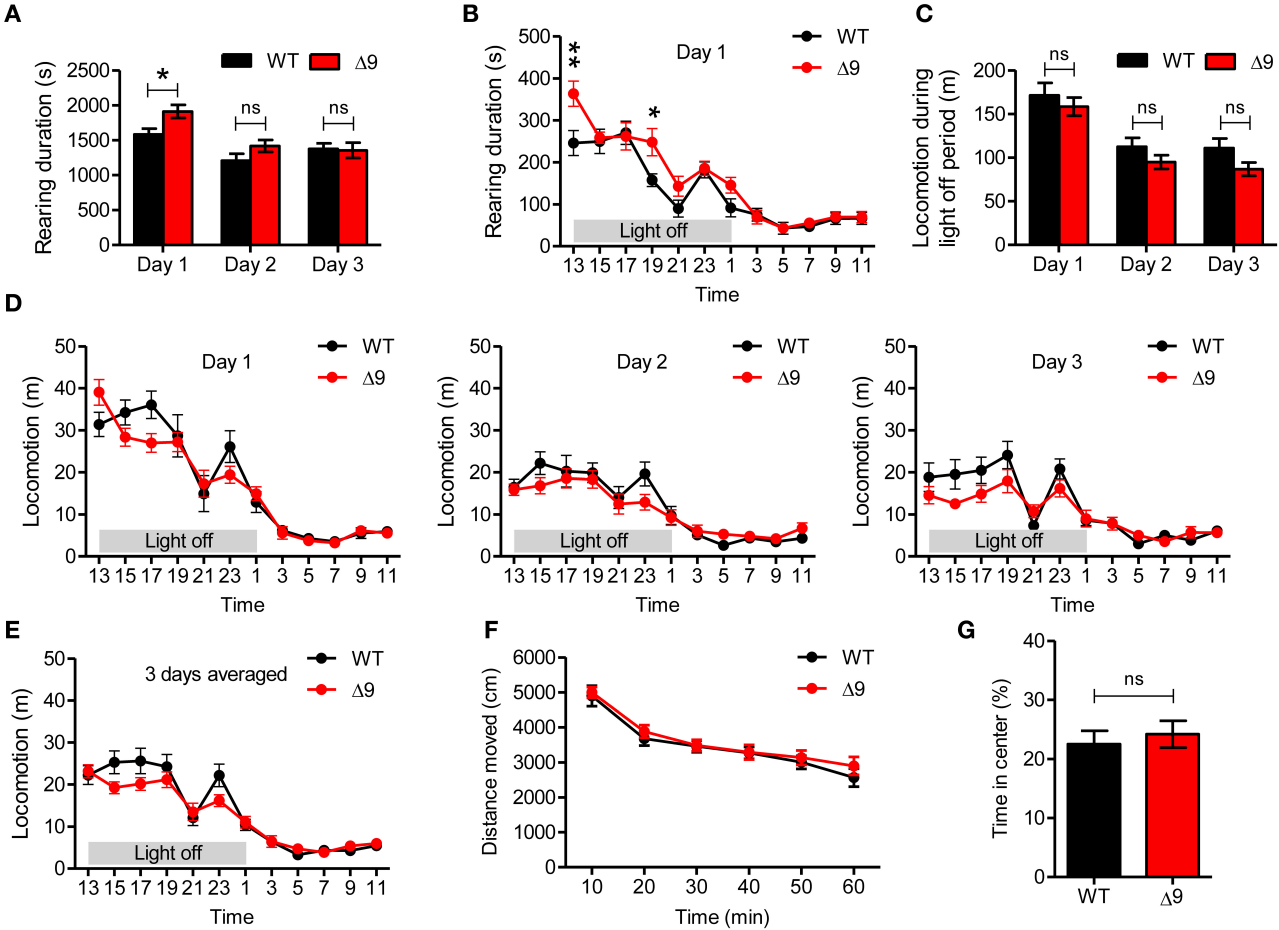
***Shank3*^Δ*9*^ mice show increased rearing but normal locomotion in a novel environment. (A)** *Shank3*^Δ*9*^ mice (2–4 months) show increased rearing on day 1 but not on days 2 or 3, as determined by 72-h consecutive monitoring of behavior. **(B)** During day 1, increased rearing was stronger during the first 2 h in a novel home-cage–like environment. **(C)** *Shank3*^Δ*9*^ mice show locomotor activities comparable to those of WT mice during light-off periods on days 1, 2, and 3. **(D, E)** *Shank3*^Δ*9*^ mice show normal levels of locomotor activities, as measured by 72-h consecutive monitoring of behavior. It should be noted that *Shank3*^Δ*9*^ mice tended to be hyperactive during the first 2 h on day 1, but were hypoactive during the rest of day 1 and two following days (days 2 and 3) **(D)**, as is also evident from locomotor activities averaged over all 3 days **(E)**. **(F, G)** *Shank3*^Δ*9*^ mice (2–4 months) show normal locomotion in the open field test **(F)** and normal time spent in the center region of the open field arena compared with WT mice **(G)**. ^*^P < 0.05, ^**^P < 0.01, ns, not significant, Student's *t*-test (**A, C, G**), Two-Way repeated measures ANOVA (**B–F**). Data represent mean ± standard error.

Next, we further analyzed the 72-h rearing movements of *Shank3*^Δ*9*^ mice by dissecting the movements into daily and 2-h segments. We found that *Shank3*^Δ*9*^ mice showed significantly increased rearing on day 1, but not on days 2 or 3 (Figure 5A; Table 2). In addition, the increased rearing on day 1 was most prominent during the first 2 h (Figure 5B). This suggests that *Shank3*^Δ*9*^ mice show increased rearing upon introduction to a novel environment.

We then analyzed whether *Shank3*^Δ*9*^ mice exhibited altered horizontal locomotion. We found that the locomotor activities of *Shank3*^Δ*9*^ mice (2–4 months) were not significantly different from those of WT mice on days 1, 2, or 3 (Figure 5C). In addition, locomotion during the first 2-h period on day 1 was not different between genotypes, although there was a strong tendency toward an increase in *Shank3*^Δ*9*^ mice (Figure 5D). Notably, after the initial 2 h, *Shank3*^Δ*9*^ mice tended to be less active than WT mice, a tendency that continued through the second and third days (Figure 5D). This hypoactive tendency was also evident when movements were averaged over all 3 days (Figure 5E).

Lastly, we measured the locomotion of *Shank3*^Δ*9*^ mice (2–4 months) for 60 min in an open field test, which represents a novel environment. We found no difference between genotypes in locomotor activity (Figure 5F), result similar to that observed in the first 2 h of the 72-h measurement (Figure 5D). In addition, *Shank3*^Δ*9*^ mice spent a normal amount of time in the center region of the open field arena (Figure 5G), suggesting the absence of anxiety-like behavior. These results, together with the results from 72-h measurements of locomotion, suggest that locomotion in both novel and familiar environments is normal in *Shank3*^Δ*9*^ mice.

### *Shank3*^Δ*9*^ mice show mildly impaired spatial memory

Although *Shank3*^Δ*9*^ mice did not show autistic-like behaviors, we reasoned that the decreased E/I ratio in the hippocampal CA1 region might be associated with changes in hippocampal function. To test this, we subjected *Shank3*^Δ*9*^ mice to the Morris water maze, a behavioral paradigm known to measure hippocampus-dependent spatial learning and memory (Morris, 1984).

**Figure 6:**
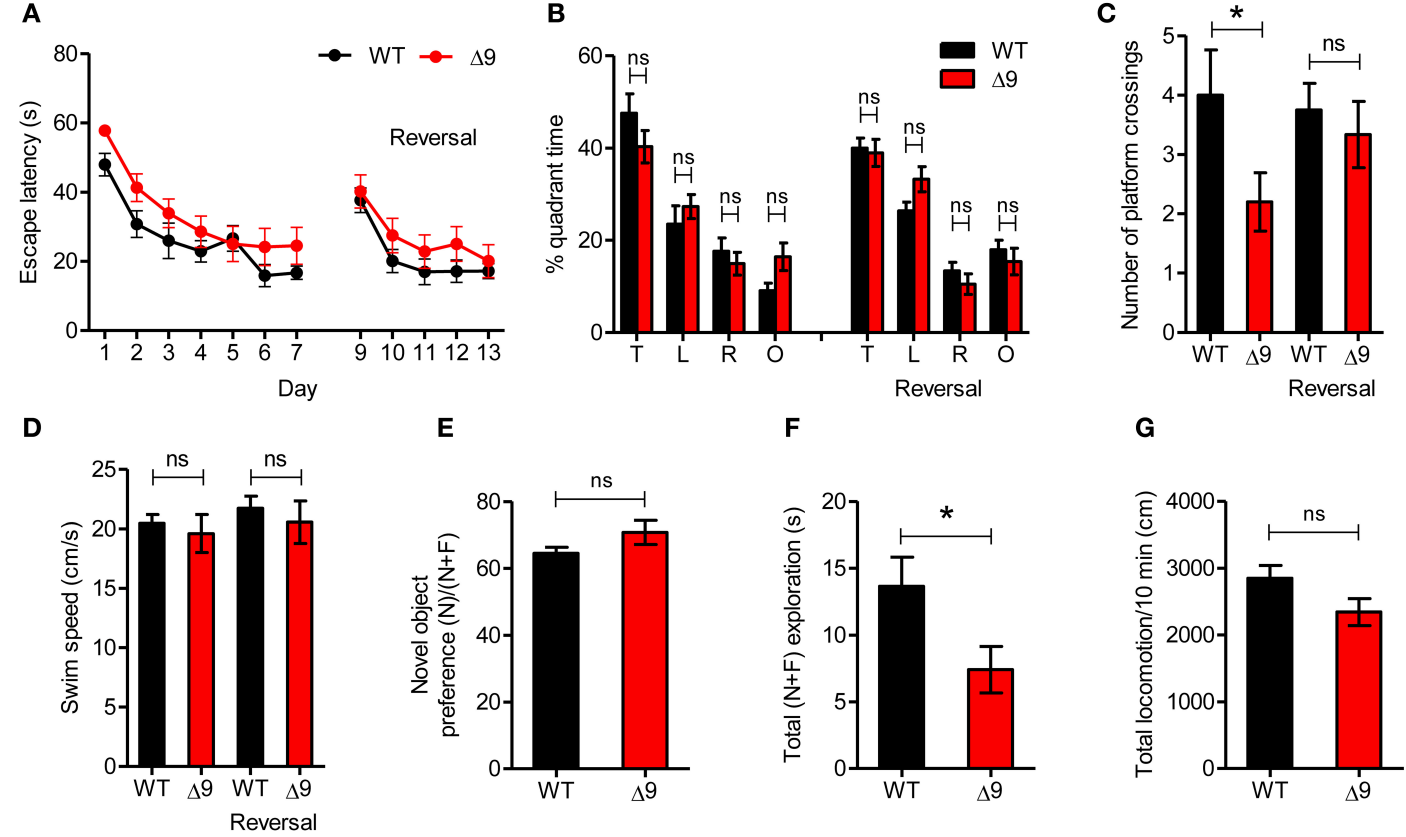
***Shank3*^Δ*9*^mice show mildly impaired spatial memory. (A)** *Shank3*^Δ*9*^ mice (2–4 months) perform normally in the learning and reversal-learning phases of the Morris water maze test, as measured by time taken to escape to platforms. *N* = 12 (WT), 15 (*Shank3*^Δ*9*^). Not significant at all-time points (not indicated), Two-Way repeated measures ANOVA. **(B, C)** *Shank3*^Δ*9*^ mice show normal quadrant occupancy **(B)** but decreased number of crossings over the platform location **(C)** during the probe phase of the Morris water maze test. *N* = 12 (WT), 15 (*Shank3*^Δ*9*^). **(D)** Swim speeds in the Morris water maze were similar between genotypes. *N* = 12 (WT), 15 (*Shank3*^Δ*9*^). **(E–G)** *Shank3*^Δ*9*^ mice (2–4 months) show normal novel-object preference in the novel object recognition test **(E)**, although total time spent exploring the objects was reduced **(F)**; total locomotion during the test phase was unaltered **(G)**. *N* = 12 (WT), 15 (*Shank3*^Δ*9*^). ^*^P < 0.05, ns, not significant, Student's *t*-test. Data represent mean ± standard error.

We found that *Shank3*^Δ*9*^ mice (2–4 months) performed normally during the learning phase of the Morris water maze test (Figure 6A; Table 2). In addition, target quadrant-occupancy scores for *Shank3*^Δ*9*^ mice were comparable to those of WT mice in the probe test (Figure 6B). However, *Shank3*^Δ*9*^ mice showed a reduced number of exact platform crossings (Figure 6C), a more stringent measure of spatial memory. *Shank3*^Δ*9*^ mice showed a normal swimming speed (Figure 6D). When mice were subjected to the reversal-learning paradigm in the Morris water maze, *Shank3*^Δ*9*^ mice performed normally during the reversal learning and probe phases, including exact platform crossings (Figures 6A–C). Collectively, these results suggest that *Shank3*^Δ*9*^ mice have a mild impairment in spatial memory.

Next, to measure recognition memory, we subjected *Shank3*^Δ*9*^ mice to a novel object recognition test in which a mouse familiarized to two identical objects on the day 1 is exposed to a novel object that replaces one of the two familiar objects on day 2 (Ennaceur and Delacour, 1988). We found that *Shank3*^Δ*9*^ mice (2–4 months) showed normal levels of novel object recognition, compared with WT mice (Figure 6E). Notably, *Shank3*^Δ*9*^ mice spent less time exploring objects on day 2 (Figure 6F), despite their normal level of locomotor activity (Figure 6G), suggesting that *Shank3*^Δ*9*^ mice have a tendency toward reduced exploration of objects in a familiar environment. Together, these results suggest that *Shank3*^Δ*9*^ mice display mildly impaired spatial memory and tend to be hypoactive in a familiar environment.

## Discussion

In the present study, we explored the effect of *Shank3* exon-9 deletion in mice. Exon 9 is predicted to be included in five of the 10 splice variants of Shank3 (Wang et al., 2014b). We first sought to identify specific Shank3 protein variants that are eliminated in the *Shank3*^Δ*9*^ brain using three independent Shank3 antibodies. However, this goal could not be fully achieved because the available antibodies did not recognize all of the spliced regions. In addition, bands of smaller-sized proteins could not readily be distinguished from products of degradation or modification. Despite these practical difficulties, immunoblot analyses clearly revealed that Shank3a, the longest Shank3 protein variant, was absent in the *Shank3*^Δ*9*^ brain (Figures 1D, 2D).

Using X-gal staining, which provides a stronger signal-to-noise ratio than antibody staining, we were able to clearly visualize brain regions that were positive for ankyrin repeat-containing Shank3 protein variants. These regions included the cortex, striatum, hippocampus, and thalamus. The strong signals in these regions sharply contrasted with the apparent lack of signals in other brain regions, including the olfactory bulb, hypothalamus, midbrain, cerebellum, pons, and medulla. Therefore, our X-gal staining data establish clear and distinct spatial expression patterns of ankyrin repeat-containing Shank3 protein variants. In addition, these results predict that the deletion of *Shank3* exon 9 in mice will likely have differential impacts in different brain regions.

X-gal staining also revealed differential expression within a given brain region. For instance, Shank3 protein signals were stronger in cortical layers 2–4 than in layers 5 and 6, and in the CA1 and DG subfields of the hippocampus relative to the CA3 region (Figure 2C; Supplementary Figures 1, 2). The stronger Shank3 expression in upper cortical layers is reminiscent of the reported enrichment of ASD risk gene modules (i.e., *SHANK2*, *CNTNAP2*, *NRXN1*, and *NLGN1*) in the superficial layers (layers 2–4) of the human cortex (Parikshak et al., 2013).

The lack of ankyrin repeat-containing Shank3 proteins in the cerebellum and olfactory bulb revealed by X-gal staining sharply contrasts with the strong expression of the corresponding mRNAs in these regions (Figures 2B,C). Therefore, care should be taken in interpreting spatiotemporal and activity-dependent changes in the expression patterns of Shank3 mRNAs, as they may not reflect the actual changes at the protein level. This observation also points to the possibility that Shank3 mRNA variants might be regulated during their translation into proteins, which is in line with the presence of Shank3 mRNAs in dendritic/axonal compartments, and their relatively short half-lives (18–28 h) (Epstein et al., 2014).

*Shank3*^Δ*9*^ SC-CA1 synapses displayed reduced excitatory synaptic transmission, as measured by the input-output relationship, but showed normal LTP (Figures 3A–C). The reduction in excitatory transmission is similar to the previously reported reduced input-output relationship observed at SC-CA1 synapses of Shank3 mutant mice lacking exons 4–9 (termed *Shank3*^*e4-9*^ hereafter) (Yang et al., 2012), but unlike the normal input-output relationship observed in another *Shank3*^*e4-9*^ mouse line (Wang et al., 2011). Although it is not clear how the deletion of the same exons 4–9 causes different changes in input-output relationships, these results, together with ours, indicate that ankyrin repeat-containing Shank3 variants may be important for the maintenance of excitatory synaptic strength. The lack of changes in LTP at *Shank3*^Δ*9*^ SC-CA1 synapses was somewhat unexpected given the reduced LTP observed at the same synapses in *Shank3*^*e4-9*^ mice (Yang et al., 2012). This apparent discrepancy might be attributable to the difference in the LTP-inducing stimulus: a high-frequency stimulation in our study and theta burst stimulation in the previous study (Yang et al., 2012).

*Shank3*^Δ*9*^ mice displayed increased mIPSC frequency in CA1 pyramidal neurons (Figure 3E), an observation that was unexpected because Shank proteins are mainly located at excitatory synapses but not inhibitory synapses (Boeckers et al., 1999; Naisbitt et al., 1999; Lim et al., 1999; Tu et al., 1999; Yao et al., 1999; Valtschanoff and Weinberg, 2001; Petralia et al., 2005). Given that changes in the frequency of spontaneous synaptic transmission often reflect changes in presynaptic terminals or neurons, and the fact that Shank3 proteins are expressed in medium spiny neurons in the striatum (Peca et al., 2011), it is possible that deletion of *Shank3* exon 9 might cause a change in GABAergic neurons that synapse onto excitatory postsynaptic neurons.

*Shank3*^Δ*9*^ mice displayed decreased mIPSC frequency in mPFC pyramidal neurons (Figure 3G), a change that contrasts with the increased mIPSC frequency observed in the hippocampus. This change likely increases the E/I ratio, and might also involve changes in presynaptic GABAergic neurons synapsing onto layer 2/3 pyramidal neurons. Although the underlying mechanisms remain to be further studied, similar results—distinct electrophysiological changes in different brain regions induced by the disruption of a single ASD-related gene—have been reported. For instance, mice lacking the excitatory synaptic scaffolding protein IRSp53 show enhanced NMDA receptor-mediated synaptic transmission and normal mEPSCs in the hippocampus but normal NMDA receptor transmission and decreased mEPSC frequency and amplitude in the mPFC (Chung et al., 2015). In addition, *Neuroligin-3*^R451C^ knock-in mice, expressing an ASD-related mutation found in humans, show enhanced inhibitory synaptic transmission in the somatosensory cortex, but enhanced NMDAR function in the hippocampus (Tabuchi et al., 2007; Etherton et al., 2011). In addition, inhibitory input onto CA1 pyramidal neurons from parvalbumin- and cholecystokinin-expressing basket cells is decreased and increased, respectively (Foldy et al., 2013). Furthermore, inhibitory input onto D1 dopamine receptor-containing neurons in the nucleus accumbens is inhibited (Rothwell et al., 2014). Therefore, the same neuroligin-3 mutation leads to diverse electrophysiological phenotypes in distinct brain regions and circuits.

The altered E/I ratio in distinct *Shank3*^Δ*9*^ brain regions is intriguing from the pathophysiological point of view. An increase in the E/I ratio in mPFC pyramidal neurons by optogenetic stimulation has been shown to induce social and memory deficits and high-frequency cortical oscillations in mice, which are observed in individuals with ASDs and schizophrenia, and the optogenetically induced social deficits are improved by enhancing inhibitory drive from palvalbumin-positive GABAergic interneurons synapsing onto pyramidal neurons (Yizhar et al., 2011). In addition, a disturbed E/I balance has been observed in animal models of ASDs (Rubenstein and Merzenich, 2003; Hines et al., 2008; Sudhof, 2008; Gogolla et al., 2009; LeBlanc and Fagiolini, 2011; Pizzarelli and Cherubini, 2011; Gandal et al., 2012; Gkogkas et al., 2013; Lin et al., 2013; Tyzio et al., 2014), and is associated with diverse neuropsychiatric and neurological disorders, including ASDs, intellectual disability, epilepsy, and schizophrenia (Eichler and Meier, 2008; Marin, 2012). Therefore, the altered E/I balance in *Shank3*^Δ*9*^ mice might underlie some of the behavioral abnormalities of these mice including enhanced rearing and mildly impaired spatial memory.

*Shank3*^Δ*9*^ mice show enhanced rearing in a novel environment (Figures 5A,B). Enhanced rearing has been thought to involve emotionality, in addition to explorative activity (Gorisch and Schwarting, 2006). However, *Shank3*^Δ*9*^ mice did not show altered anxiety-like behavior, as shown by the normal time spent in the center region of the open field arena. Mechanisms underlying the enhanced rearing in *Shank3*^Δ*9*^ mice would be a subject for future investigations.

*Shank3*^Δ*9*^ mice show mildly impaired spatial memory in the Morris water maze (Figures 6A–C), displaying a significantly reduced number of exact platform crossings during the probe phase without a change in quadrant occupancy. This partial loss of spatial learning and memory in *Shank3*^Δ*9*^ mice is similar to the reported behavior of *Shank3*^*e4-9*^ mice, which show modestly reduced performance in the learning and probe phases of the maze (Wang et al., 2011), although exact platform crossings were not measured in this latter report. Another report, however, showed that *Shank3*^*e4-9*^ mice perform normally in the Morris water maze based on all parameters, including platform crossings (Yang et al., 2012). These results collectively suggest that the loss of ankyrin repeat-containing Shank3 proteins leads to no or partial impairments in hippocampus-dependent spatial learning and memory.

In conclusion, our data suggest that ankyrin repeat-containing variants of Shank3 are important for E/I balance, rearing behavior, and spatial memory.

### Conflict of interest statement

The authors declare that the research was conducted in the absence of any commercial or financial relationships that could be construed as a potential conflict of interest.

## Supplementary material

Supplementary Table 1 **Statistical results of the electrophysiology and behavior experiments**.

Supplementary Figure 1 **Distribution patterns of ankyrin repeat-containing Shank3 protein variants in the mouse brain**. Coronal sections of the *Shank3*^+/β−*gal*^ brain (6–7 weeks) stained with X-gal.

Supplementary Figure 2 **Presence of ankyrin repeat-containing Shank3 protein variants in upper cortical layers (layers 2–4)**. Coronal sections of the *Shank3*^+/β−*gal*^ brain (6–7 weeks) were stained by X-gal.
