# Supplementary material for: Shank3-mutant mice lacking exon 9 show altered excitation/inhibition balance, enhanced rearing, and spatial memory deficit
Source: Front Cell Neurosci. 2015 Mar 19;9:94. doi: 10.3389/fncel.2015.00094 (PMC4365696; doi:10.3389/fncel.2015.00094)
Supplement: Supplementary Table 1 — Statistical results of the electrophysiology and behavior experiments. [file Table1.DOCX]

**Supplementary Table 1. Statistical Results**

| **Figure** | **Panel** | **Assay** | **N & Age** | **Parameter** | **Mean ± SEM** | **Statistical Test and Significance** | **Conclusion** |
| --- | --- | --- | --- | --- | --- | --- | --- |
| 3 | A | Electrophysiology (Local field current clamp) | WT=9 from 3 mice  ∆9=9 from 3 mice  P19-25 | Field EPSP slope (mV/ms) against fiber volley amplitude (mV) | WT  0.05: 0.11 ± 0.01  0.1: 0.19 ± 0.01  0.15: 0.27 ± 0.02  0.2: 0.36 ± 0.03  ∆9  0.05: 0.08 ± 0.01  0.1: 0.14 ± 0.01  0.15: 0.19 ± 0.01  0.2: 0.24 ± 0.02 | Student’s t-test (WT vs ∆9)  0.05: p=0.0438  0.1: p=0.0017  0.15: p=0.0038  0.2: p=0.0069 | Synaptic transmission in hippocampal CA1 field:  WT>∆9 |
|  | B |  |  | Paired pulse ratio against inter-stimulus interval (ms) | WT  25: 2.23 ± 0.10  50: 2.32 ± 0.09  75: 2.26 ± 0.07  100: 2.06 ± 0.05  200: 1.71 ± 0.04  300: 1.46 ± 0.05  ∆9  25: 2.35 ± 0.05  50: 2.37 ± 0.07  75: 2.24 ± 0.05  100: 2.06 ± 0.05  200: 1.66 ± 0.05  300: 1.47 ± 0.03 | Student’s t-test (WT vs ∆9)  25: p=0.2673  50: p=0.6827  75: p=0.7912  100: p=0.9911  200: p=0.4184  300: p=0.8007 | Presynaptic release probability in hippocampal CA1 field:  WT=∆9 |
|  | C |  | WT=8 from 4 mice  ∆9=7 from 3 mice  P21-24 | fEPSP slope (%) before and after LTP induction | WT  -20 min: 100.84 ± 0.62  -10 min: 99.74 ± 0.77  10 min: 133.52 ± 4.83  20 min: 130.01 ± 4.50  30 min: 124.78 ± 4.93  40 min: 123.00 ± 5.32  50 min: 121.63 ± 4.98  60 min: 117.62 ± 4.52  ∆9  -20 min: 99.54 ± 1.09  -10 min: 100.82 ± 0.85  10 min: 127.01 ± 4.47  20 min: 122.90 ± 4.11  30 min: 119.43 ± 5.79  40 min: 117.12 ± 5.65  50 min: 118.57 ± 7.53  60 min: 117.79 ± 7.57 | Student’s t-test (WT vs ∆9)  -20 min: p=0.3008  -10 min: p=0.3615  10 min: p=0.3458  20 min: p=0.2692  30 min: p=0.4916  40 min: p=0.4621  50 min: p=0.7337  60 min: p=0.9845 | High frequency stimulus-induced LTP:  WT=∆9 |
|  | D | Electrophysiology  (Whole cell voltage  clamp) | WT=14 from 3 mice  ∆9=15 from 3 mice  P19-22 | mEPSC frequency (Hz) and amplitude (pA) in CA1 pyramidal cells | WT  Frequency: 0.40 ± 0.04  Amplitude: 16.33 ± 0.46  ∆9  Frequency: 0.43 ± 0.06  Amplitude: 17.24 ± 0.29 | Student’s t-test (WT vs ∆9)  Frequency: p=0.6947  Amplitude: p=0.0962 | Basal excitatory transmission (CA1):  WT=∆9 |
|  | E |  | WT=8 from 4 mice  ∆9=8 from 3 mice  P23-27 | mIPSC frequency (Hz) and amplitude (pA) in CA1 pyramidal cells | WT  Frequency: 5.07 ± 0.87  Amplitude: 56.01 ± 4.86  ∆9  Frequency: 7.68 ± 0.58  Amplitude: 68.12 ± 4.31 | Student’s t-test (WT vs ∆9)  Frequency: p=0.0251  Amplitude: p=0.0830 | Basal inhibitory transmission (CA1):  WT<∆9 (frequency) |
|  | F |  | WT=13 from 3 mice  ∆9=14 from 3 mice  P39–45 | mEPSC frequency (Hz) and amplitude (pA) in mPFC layer 2/3 cells | WT  Frequency: 10.87 ± 1.06  Amplitude: 16.58 ± 0.45  ∆9  Frequency: 8.55 ± 0.94  Amplitude: 16.43 ± 0.36 | Student’s t-test (WT vs ∆9)  Frequency: p=0.1135  Amplitude: p=0.8033 | Basal excitatory transmission (mPFC):  WT=∆9 |
|  | G |  | WT=10 from 4 mice  ∆9= 10 from 3 mice  P39–54 | mIPSC frequency (Hz) and amplitude (pA) in mPFC layer 2/3 cells | WT  Frequency: 6.46 ± 0.53  Amplitude: 46.00 ± 4.14  ∆9  Frequency: 4.83 ± 0.46  Amplitude: 41.46 ± 4.53 | Student’s t-test (WT vs ∆9)  Frequency: p=0.0317  Amplitude: p=0.4696 | Basal inhibitory transmission (mPFC):  WT>∆9  (frequency) |
| 4 | A | 3-chamber social interaction (Object vs Stranger 1) | WT=23  ∆9=19  2-4 months | Exploration time (s) | WT O: 7.02 ± 0.91  WT S1: 26.41 ± 2.62  ∆9 O: 5.13 ± 0.92  ∆9 S1: 30.86 ± 4.92 | Student’s t-test  WT O vs WT S1: p< 0.0001  ∆9 O vs ∆9 S1: p< 0.0001 | Social interaction:  WT=∆9 |
|  | B |  |  | Social preference index | WT: 51.44 ± 8.60  ∆9: 60.71 ± 10.12 | Student’s t-test  WT vs ∆9: p=0.4863 |  |
|  | C | 3-chamber social interaction (Stranger 1 vs Stranger 2) | WT=8  ∆9=10  2-4 months | Exploration time (s) | WT S1: 1.32 ± 0.39  WT S2: 11.02 ± 2.88  ∆9 S1: 6.10 ± 3.57  ∆9 S2: 15.94 ± 2.69 | Student’s t-test  WT S1 vs WT S2: p=0.0048  ∆9 S1 vs ∆9 S2: p=0.0410 | Social novelty recognition:  WT=∆9 |
|  | D |  |  | Social preference index | WT: 68.63 ± 8.33  ∆9: 60.19 ± 10.91 | Student’s t-test  WT vs ∆9: p=0.5639 |  |
|  | E | Pup ultrasonic vocalization | WT=11  ∆9=9  P4~P10 | Number of USV calls/3 min | WT P4: 143.55 ± 22.94  ∆9 P4: 131.89 ± 38.66  WT P6: 193.82 ± 34.36  ∆9 P6: 251.22 ± 54.82  WT P8: 146.45 ± 26.56  ∆9 P8: 157.78 ± 26.10  WT P10: 114.45 ± 25.82  ∆9 P10: 102.22 ± 18.41 | Student’s t-test  WT P4 vs ∆9 P4: p=0.7899  WT P6 vs ∆9 P6: p=0.3693  WT P8 vs ∆9 P8: p=0.7674  WT P10 vs ∆9 P10: p= 0.7162 | USV emission in pups:  WT=∆9 |
|  | F | Behavior monitoring for 3 consecutive days with LABORAS | WT=9  ∆9=11  2-4 months | Total time (min) spent doing each behavior during 3 days | WT Climb: 639.74 ± 74.60  ∆9 Climb: 560.13 ± 70.66  WT Rear: 69.48 ± 2.62  ∆9 Rear: 78.14 ± 2.80  WT Groom: 419.14 ± 31.88  ∆9 Groom: 510.19 ± 31.35  WT Drink: 0.86 ± 0.23  ∆9 Drink: 2.17 ± 0.54  WT Eat: 151.81 ± 18.25  ∆9 Eat: 168.78 ± 20.29 | Student’s t-test  WT Climb vs ∆9 Climb: p=0.4507  WT Rear vs ∆9 Rear: p=0.0398  WT Groom vs ∆9 Groom: p=0.0587  WT Drink vs ∆9 Drink: p=0.0536  WT Eat vs ∆9 Eat: p=0.5505 | Rearing behavior during 3 days:  WT<∆9 |
| 5 | A | Behavior monitoring for 3 consecutive days with LABORAS | WT=9  ∆9=11  2-4 months | Total time (s) spent in rearing each day | WT Day 1: 1585 ± 81.0  ∆9 Day 1: 1914 ± 94.8  WT Day 2: 1208 ± 99.5  ∆9 Day 2: 1419 ± 86.0  WT Day 3: 1376 ± 80.9  ∆9 Day 3: 1356 ± 110.2 | Student’s t-test  WT Day 1 vs ∆9 Day 1: p=0.0194  WT Day 2 vs ∆9 Day 2: p=0.1248  WT Day 3 vs ∆9 Day 3: p=0.8888 | Rearing behavior in day 1:  WT<∆9 |
|  | B |  |  | Time (s) spent in rearing at different time points on day 1 | WT  13: 246.06 ± 29.64  15: 249.83 ± 28.90  17: 270.45 ± 27.70  19: 157.33 ± 15.08  21: 89.62 ± 20.18  23: 182.08 ± 18.94  1: 91.56 ± 21.54  3: 75.38 ± 15.11  5: 43.13 ± 14.03  7: 46.87 ± 10.78  9: 65.99 ± 13.89  11: 66.34 ± 14.42  ∆9  13: 363.54 ± 30.27  15: 258.71 ± 13.45  17: 261.76 ± 32.25  19: 248.32 ± 32.37  21: 142.88 ± 23.56  23: 185.66 ± 17.09  1: 145.45 ± 18.57  3: 69.20 ± 16.09  5: 43.04 ± 10.45  7: 55.60 ± 11.95  9: 69.74 ± 12.42  11: 69.66 ± 13.16 | 2 way repeated measures ANOVA  13: p<0.01  15: p>0.05  17: p>0.05  19: p<0.05  21: p>0.05  23: p>0.05  1: p>0.05  3: p>0.05  5: p>0.05  7: p>0.05  9: p>0.05  11: p>0.05 | Rearing behavior at first two hours of day 1:  WT<∆9 |
|  | C |  |  | Locomotion (m) during light off period each day | WT Day 1: 171.44 ± 14.27  ∆9 Day 1: 158.30 ± 10.52  WT Day 2: 112.36 ± 10.34  ∆9 Day 2: 94.93 ± 7.94  WT Day 3: 111.07 ± 10.90  ∆9 Day 3: 86.74 ± 7.67 | Student’s t-test  WT Day 1 vs ∆9 Day 1: p=0.4590  WT Day 2 vs ∆9 Day 2: p=0.1906  WT Day 3 vs ∆9 Day 3: p=0.0774 | Activity level during light off period:  WT=∆9 |
|  | D |  |  | Locomotion (m) at different time points on day 1, 2, 3 | Day 1  WT  13: 31.40 ± 2.87  15: 34.26 ± 2.96  17: 36.07 ± 3.25  19: 28.69 ± 5.04  21: 14.93 ± 4.26  23: 26.10 ± 3.80  1: 12.92 ± 2.45  3: 6.17 ± 1.23  5: 4.24 ± 1.02  7: 3.52 ± 0.75  9: 5.52 ± 1.27  11: 5.92 ± 1.16  ∆9  13: 39.04 ± 3.06  15: 28.38 ± 2.13  17: 26.98 ± 2.22  19: 27.20 ± 2.29  21: 17.29 ± 3.17  23: 19.42 ± 2.01  1: 14.92 ± 1.60  3: 5.56 ± 1.48  5: 3.71 ± 0.56  7: 3.20 ± 0.50  9: 6.12 ± 1.01  11: 5.54 ± 0.70  Day 2  WT  13: 16.45 ± 1.89  15: 22.15 ± 2.71  17: 20.28 ± 3.77  19: 19.87 ± 2.33  21: 14.00 ± 2.61  23: 19.60 ± 2.85  1: 9.74 ± 2.15  3: 5.12 ± 1.02  5: 2.60 ± 0.64  7: 4.36 ± 0.82  9: 3.48 ± 0.56  11: 4.32 ± 0.61  ∆9  13: 15.87 ± 1.33  15: 16.82 ± 1.93  17: 18.56 ± 2.29  19: 18.31 ± 2.08  21: 12.47 ± 2.37  23: 12.91 ± 1.84  1: 9.22 ± 1.85  3: 6.01 ± 1.46  5: 5.28 ± 1.11  7: 4.79 ± 1.07  9: 4.23 ± 1.05  11: 6.73 ± 1.24  Day 3  WT  13: 18.84 ± 3.38  15: 19.52 ± 3.52  17: 20.48 ± 3.12  19: 24.09 ± 3.25  21: 7.35 ± 1.13  23: 20.79 ± 2.37  1: 8.60 ± 1.25  3: 7.89 ± 0.92  5: 3.01 ± 0.72  7: 5.00 ± 0.82  9: 3.83 ± 0.81  11: 6.09 ± 0.85  ∆9  13: 14.55 ± 2.04  15: 12.53 ± 1.21  17: 14.86 ± 1.99  19: 17.90 ± 2.71  21: 10.74 ± 1.52  23: 16.16 ± 2.02  1: 9.00 ± 1.98  3: 7.79 ± 1.50  5: 5.01 ± 0.94  7: 3.51 ± 1.10  9: 5.72 ± 1.36  11: 5.65 ± 1.09 | 2 way repeated measures ANOVA  Day 1  13: p>0.05  15: p>0.05  17: p>0.05  19: p>0.05  21: p>0.05  23: p>0.05  1: p>0.05  3: p>0.05  5: p>0.05  7: p>0.05  9: p>0.05  11: p>0.05  Day 2  13: p>0.05  15: p>0.05  17: p>0.05  19: p>0.05  21: p>0.05  23: p>0.05  1: p>0.05  3: p>0.05  5: p>0.05  7: p>0.05  9: p>0.05  11: p>0.05  Day 3  13: p>0.05  15: p>0.05  17: p>0.05  19: p>0.05  21: p>0.05  23: p>0.05  1: p>0.05  3: p>0.05  5: p>0.05  7: p>0.05  9: p>0.05  11: p>0.05 | Activity level throughout whole day:  WT=∆9 |
|  | E |  |  | Locomotion (m) at different time points (3 days averaged) | WT  13: 22.23 ± 2.26  15: 25.31 ± 2.70  17: 25.61 ± 3.03  19: 24.22 ± 2.91  21: 12.09 ± 1.87  23: 22.16 ± 2.71  1: 10.42 ± 1.32  3: 6.39 ± 0.71  5: 3.28 ± 0.49  7: 4.29 ± 0.49  9: 4.28 ± 0.68  11: 5.44 ± 0.43  ∆9  13: 23.15 ± 1.55  15: 19.24 ± 1.41  17: 20.13 ± 1.52  19: 21.14 ± 1.89  21: 13.50 ± 2.03  23: 16.16 ± 1.40  1: 11.05 ± 1.39  3: 6.45 ± 1.37  5: 4.67 ± 0.73  7: 3.83 ± 0.68  9: 5.36 ± 0.89  11: 5.97 ± 0.86 | 2 way repeated measures ANOVA  13: p>0.05  15: p>0.05  17: p>0.05  19: p>0.05  21: p>0.05  23: p>0.05  1: p>0.05  3: p>0.05  5: p>0.05  7: p>0.05  9: p>0.05  11: p>0.05 | Activity level throughout whole day:  WT=∆9 |
|  | F | Open field | WT=12  ∆9=15  2-4 months | Distance moved (cm) at different time points during 1 hour | WT  10: 4899 ± 292.79  20: 3677 ± 196.04  30: 3458 ± 167.59  40: 3271 ± 173.77  50: 3002 ± 188.11  60: 2562 ± 259.06  ∆9  10: 4999 ± 152.96  20: 3882 ± 181.02  30: 3486 ± 162.33  40: 3288 ± 208.29  50: 3135 ± 206.77  60: 2896 ± 258.28 | 2 way repeated measures ANOVA  10: p>0.05  20: p>0.05  30: p>0.05  40: p>0.05  50: p>0.05  60: p>0.05 | Activity level during 1 hour in a novel environment:  WT=∆9 |
|  | G |  |  | % time spent in center zone of open field | WT: 22.50 ± 2.26  ∆9: 24.18 ± 2.29 | Student’s t-test  WT vs ∆9: p= 0.6115 | Anxiety level in a novel, open environment:  WT=∆9 |
| 6 | A | Morris water maze | WT=12  ∆9=15  2-4 months | Escape latency (s) in learning & reversal learning phases | WT  Day 1: 47.92 ± 3.23  Day 2: 30.75 ± 3.85  Day 3: 25.92 ± 5.11  Day 4: 22.89 ± 3.07  Day 5: 26.61 ± 3.73  Day 6: 15.86 ± 3.17  Day 7: 16.69 ± 1.91  Day 9: 37.61 ± 3.56  Day 10: 20.06 ± 3.34  Day 11: 16.94 ± 3.70  Day 12: 17.11 ± 3.24  Day 13: 17.17 ± 2.18  ∆9  Day 1: 57.73 ± 1.56  Day 2: 41.27 ± 4.00  Day 3: 33.84 ± 4.13  Day 4: 28.53 ± 4.55  Day 5: 24.98 ± 5.09  Day 6: 24.16 ± 5.37  Day 7: 24.49 ± 5.34  Day 9: 40.16 ± 4.79  Day 10: 27.44 ± 4.99  Day 11: 22.82 ± 4.83  Day 12: 25.02 ± 4.99  Day 13: 20.07 ± 4.75 | 2 way repeated measures ANOVA  Day 1: p>0.05  Day 2: p>0.05  Day 3: p>0.05  Day 4: p>0.05  Day 5: p>0.05  Day 6: p>0.05  Day 7: p>0.05  Day 9: p>0.05  Day 10: p>0.05  Day 11: p>0.05  Day 12: p>0.05  Day 13: p>0.05 | Spatial learning:  WT=∆9 |
|  | B |  |  | % time spent in each quadrant in probe test | WT  Target: 47.57 ± 4.20  Left: 23.52 ± 3.95  Right: 17.59 ± 2.92  Opposite: 9.08 ± 1.64  ∆9  Target: 40.30 ± 3.52  Left: 27.30 ± 2.61  Right: 14.92 ± 2.49  Opposite: 16.42 ± 3.00  <Reversal>  WT  Target: 40.02 ± 2.18  Left: 26.37 ± 1.94  Right: 13.39 ± 1.85  Opposite: 17.93 ± 2.08  ∆9  Target: 38.96 ± 2.95  Left: 33.24 ± 2.70  Right: 10.47 ± 2.22  Opposite: 15.39 ± 2.89 | Student’s t-test  WT Target vs ∆9 Target: p=0.1931  WT Left vs ∆9 Left: p=0.4155  WT Right vs ∆9 Right: p=0.4913  WT Opposite vs ∆9 Opposite: p=0.0562  <Reversal>  WT Target vs ∆9 Target: p=0.7863  WT Left vs ∆9 Left: p=0.0603  WT Right vs ∆9 Right: p=0.3376  WT Opposite vs ∆9 Opposite: p=0.5014 | Spatial learning:  WT=∆9 |
|  | C |  |  | Number of platform area crossings during probe test | WT: 4.00 ± 0.76  ∆9: 2.20 ± 0.49  <Reversal>  WT: 3.75 ± 0.45  ∆9: 3.33 ± 0.56 | Student’s t-test  WT vs ∆9: p=0.0492  <Reversal>  WT vs ∆9: p=0.5791 | Exactness of spatial learning:  WT>∆9 |
|  | D |  |  | Swim speed (cm/s) | WT: 20.45 ± 0.75  ∆9: 19.60 ± 1.59  <Reversal>  WT: 21.72 ± 1.02  ∆9: 20.56 ± 1.80 | Student’s t-test  WT vs ∆9: p=0.6597  <Reversal>  WT vs ∆9: p=0.6031 | Swimming ability:  WT=∆9 |
|  | E | Novel object recognition | WT=12  ∆9=15  2-4 months | Novel object preference index | WT: 64.50 ± 1.81  ∆9: 70.77 ± 3.62 | Student’s t-test  WT vs ∆9: p=0.1633 | Novel object recognition:  WT=∆9 |
|  | F |  |  | Total (novel + familiar) exploration time (s) | WT: 13.65 ± 2.17  ∆9: 7.40 ± 1.74 | Student’s t-test  WT vs ∆9: p=0.0315 | Absolute exploration time:  WT>∆9 |
|  | G |  |  | Total locomotion (cm) during 10 min exploration | WT: 2848 ± 194.40  ∆9: 2343 ± 200.50 | Student’s t-test  WT vs ∆9: p= 0.0875 | Activity level during exploration period:  WT=∆9 |
