# Supplementary figures and images for: Shank3-mutant mice lacking exon 9 show altered excitation/inhibition balance, enhanced rearing, and spatial memory deficit
Source: Front Cell Neurosci. 2015 Mar 19;9:94. doi: 10.3389/fncel.2015.00094 (PMC4365696; doi:10.3389/fncel.2015.00094)

Supplementary Figure 1

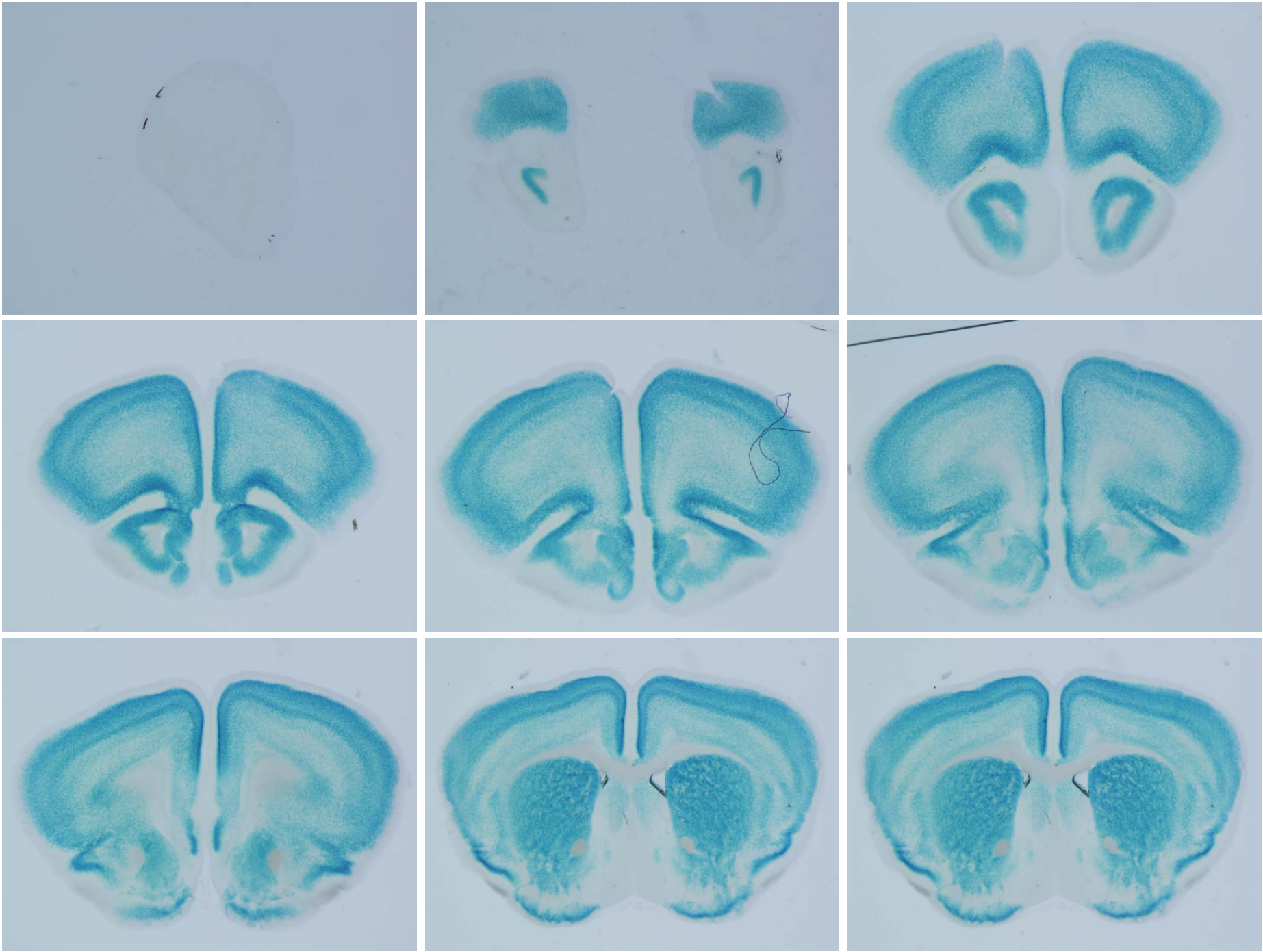

Supplementary Figure 1

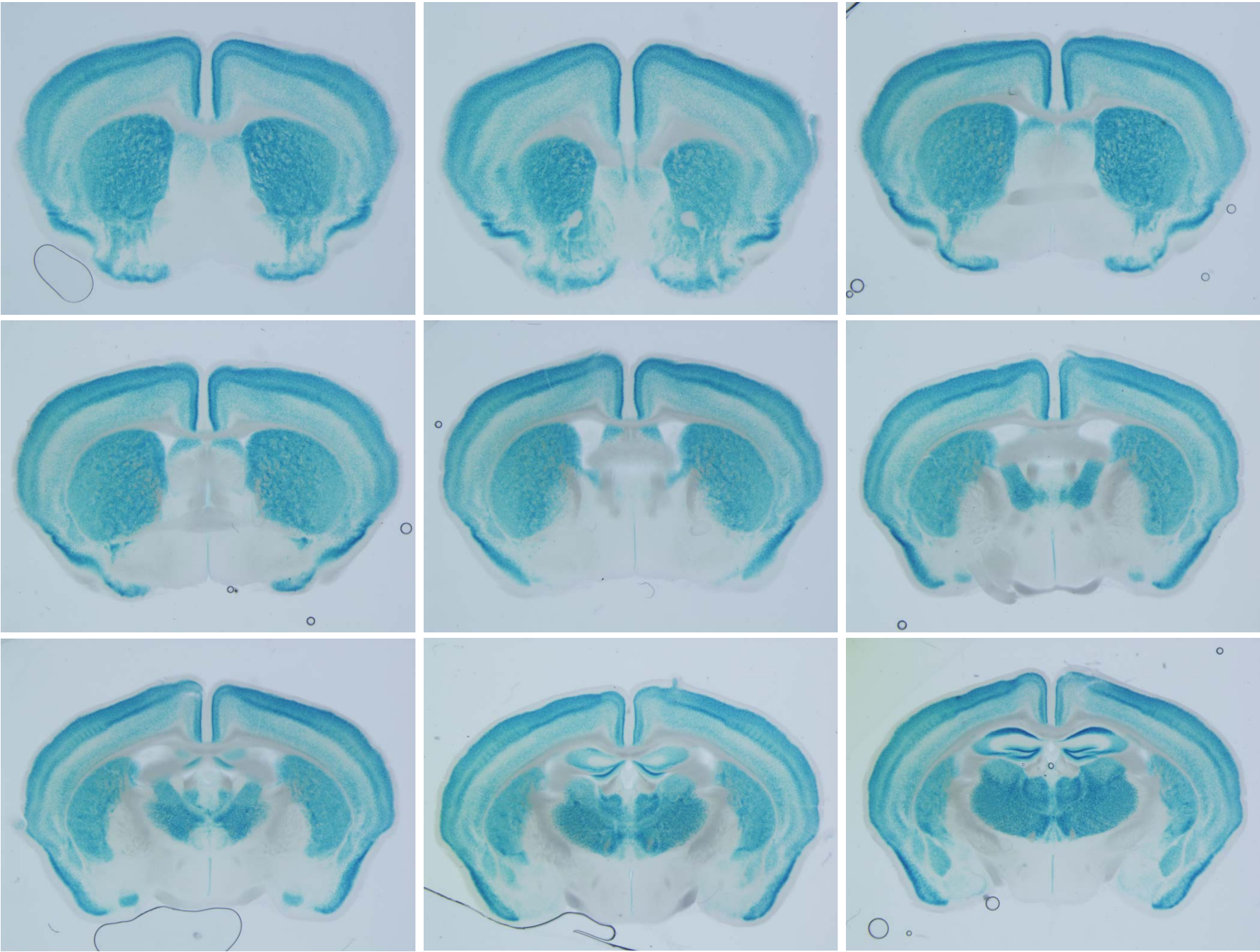

**Supplementary Figure 1**

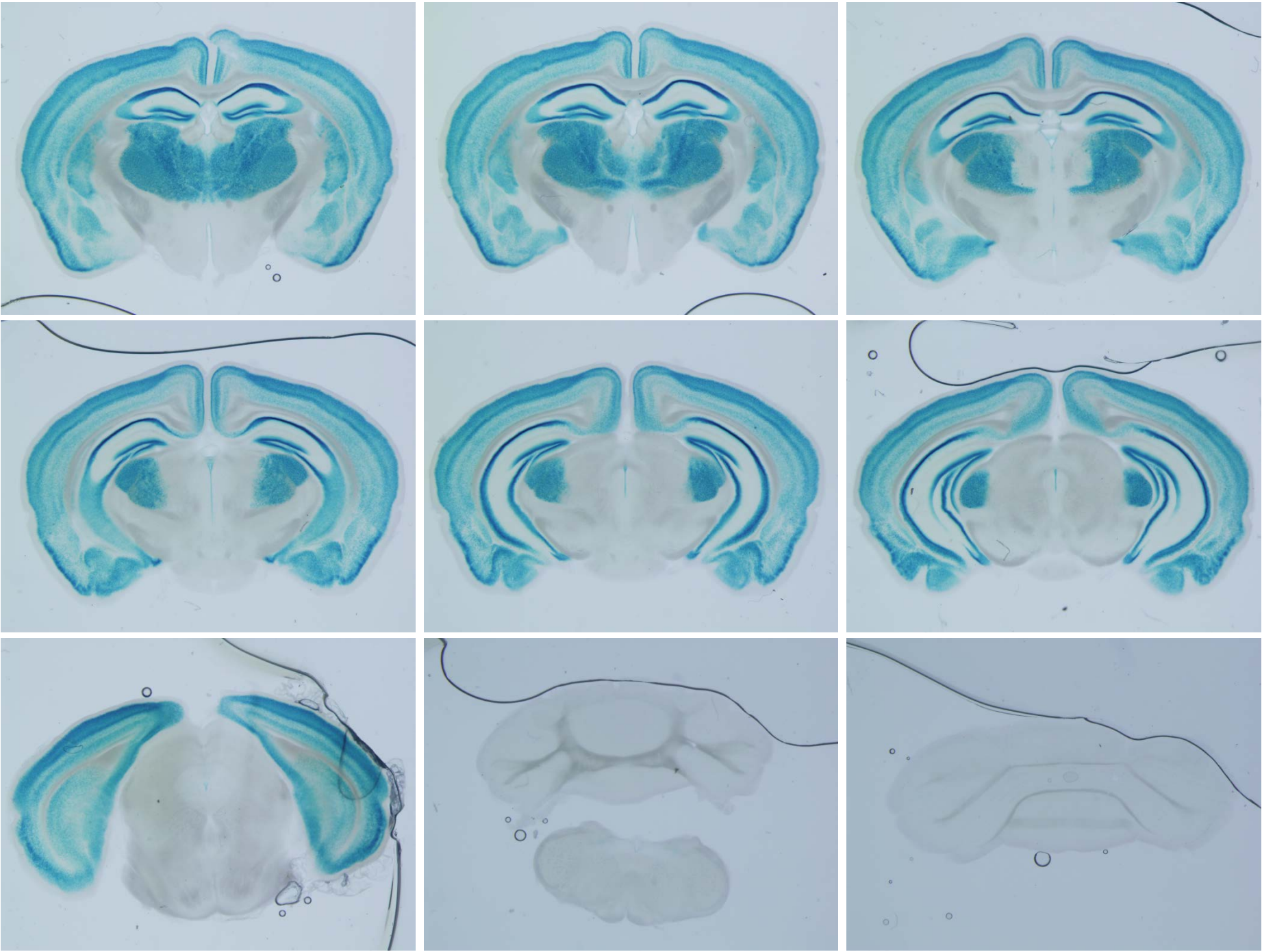

Supplementary Figure 2

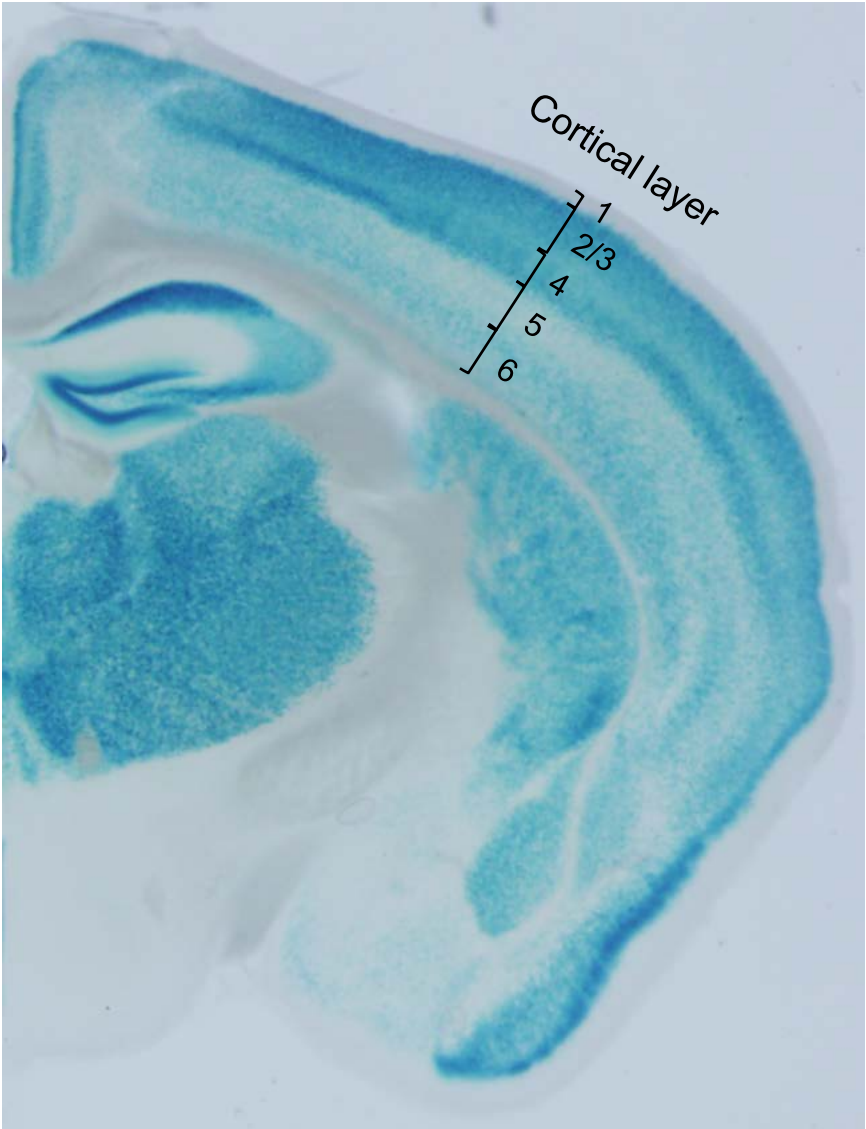

Supplement: Supplementary Figure 1 — Distribution patterns of ankyrin repeat-containing Shank3 protein variants in the mouse brain. Coronal sections of the Shank3+/β−gal brain (6–7 weeks) stained with X-gal. [file Image1.PDF]
